# Cascaded-Recalibrated Multiple Instance Deep Model for Pathologic-Level Lung Cancer Prediction in CT Images

**DOI:** 10.1155/2022/9469234

**Published:** 2022-06-13

**Authors:** Qingfeng Wang, Ying Zhou, Jun Huang, Zhiqin Liu, Weidong Zhang, Qiyu Liu, Jie-Zhi Cheng

**Affiliations:** ^1^School of Computer Science and Technology, Southwest University of Science and Technology, Mianyang, China; ^2^Department of Radiology, Mianyang Central Hospital, School of Medicine, University of Electronic Science and Technology of China, Mianyang, China; ^3^NHC Key Laboratory of Nuclear Technology Medical Transformation (Mianyang Central Hospital), Mianyang, China; ^4^Shanghai United Imaging Intelligence Co. Ltd, Shanghai, China

## Abstract

Lung cancer accounts for the greatest number of cancer-related mortality, while the accurate evaluation of pulmonary nodules in computed tomography (CT) images can significantly increase the 5-year relative survival rate. Despite deep learning methods that have recently been introduced to the identification of malignant nodules, a substantial challenge remains due to the limited datasets. In this study, we propose a cascaded-recalibrated multiple instance learning (MIL) model based on multiattribute features transfer for pathologic-level lung cancer prediction in CT images. This cascaded-recalibrated MIL deep model incorporates a cascaded recalibration mechanism at the nodule level and attribute level, which fuses the informative attribute features into nodule embeddings and then the key nodule features can be converged into the patient-level embedding to improve the performance of lung cancer prediction. We evaluated the proposed cascaded-recalibrated MIL model on the public Lung Image Database Consortium and Image Database Resource Initiative (LIDC-IDRI) benchmark dataset and compared it to the latest approaches. The experimental results showed a significant performance boost by the cascaded-recalibrated MIL model over the higher-order transfer learning, instance-space MIL, and embedding-space MIL models and the radiologists. In addition, the recalibration coefficients of the nodule and attribute feature for the final decision were also analyzed to reveal the underlying relationship between the confirmed diagnosis and its highly-correlated attributes. The cascaded recalibration mechanism enables the MIL model to pay more attention to those important nodules and attributes while suppressing less-useful feature embeddings, and the cascaded-recalibrated MIL model provides substantial improvements for the pathologic-level lung cancer prediction by using the CT images. The identification of the important nodules and attributes also provides better interpretability for model decision-making, which is very important for medical applications.

## 1. Background

Lung cancer accounts for the greatest number of cancer-related mortality, far more than breast cancer, prostate cancer, and colorectal cancer combined [1]. The 5-year relative survival rate of lung cancer is 6% when diagnosed with metastatic disease, while early diagnosis in localized stage disease can increase the survival to 59% [2]. The Multicentric Italian Lung Detection trial has reported a 39% reduction in lung cancer mortality through annual screening with low-dose CT for earlier diagnoses compared with no intervention [3]. Accordingly, the early diagnosis of lung cancer is very crucial to achieving a favorable prognosis.

**Figure 1:**
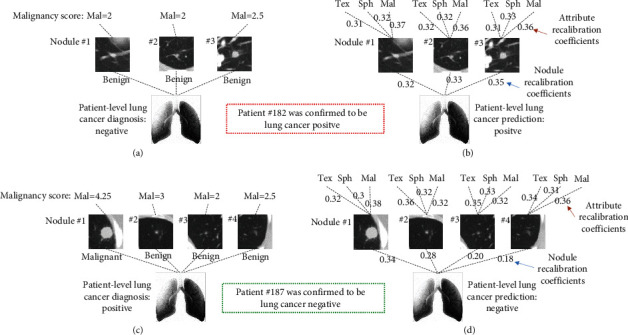
Illustration of patient-level decision-making for the lung cancer diagnosis with multiple nodules. (a) Patient #182 was diagnosed as negative by radiologists but was eventually confirmed as positive. (b) Patient #182 was predicted to be lung cancer positive by the proposed cascaded-recalibrated MIL deep model. (c) Patient #187 was diagnosed as positive by radiologists but was confirmed to be negative by surgical resection. (d) Patient #187 was predicted to be lung cancer negative by the proposed cascaded-recalibrated MIL deep model. “Tex,” “Sph,” and “Mal” are the abbreviations of semantic attributes texture, sphericity, and malignancy, respectively. (a) Patient #182 was diagnosed as lung cancer negative. (b) Patient #182 was predicted to be lung cancer positive. (c) Patient #187 was diagnosed as lung cancer positive. (d) Patient #187 was predicted to be lung cancer negative.

As lung cancer mostly develops from small pulmonary nodules, early lung cancer can be screened by analyzing the malignancy of the nodules [4–6]. In general, several nodules can be found on the chest CT scan of a patient, some of which might be malignant or benign [7]. If a patient has at least one malignant nodule, this patient should be diagnosed as lung cancer positive; a negative diagnosis of lung cancer can be made if and only if all nodules of a patient are benign. Therefore, the final decision-making for early lung cancer diagnosis shall be at the patient level, i.e., patient-level lung cancer diagnosis, which is a typical multi-instance task [8]. As shown in Figures 1(a) and 1(c), patients #182 and #187 from the LIDC-IDRI dataset [9] demonstrate the decision-making process of a patient-level lung cancer diagnosis with multiple nodules by radiologists based on the concept of a multi-instance task. Patient #182 had three nodules, all of which were assessed to be benign by radiologists. Thus, patient #182 case was diagnosed with patient-level lung cancer negative at the subjective decision making. Patient #187 had four nodules, only one of which was subjectively malignant. According to the presence of at least one malignant nodule, radiologists diagnosed patient #187 to be patient-level lung cancer positive.

In clinical practice, the qualitative assessment of the nodules mainly involves two stages of decision making: (1) subjective determination, radiologists judge whether nodules are benign or malignant by observing the morphological characteristics of nodules in CT images; (2) pathologic confirmation, nodules that are highly suspected to be malignant can be further confirmed by pathological examination like biopsy and surgical resection [10]. In fact, both patients #182 and #187 were misclassified by radiologists. Patient #182, who was subjectively diagnosed as negative by radiologists, was confirmed to be positive definitely, and patient #187, who was subjectively determined as positive by radiologists, was corrected to be negative by surgical resection. For the two stages of decision making on nodules assessment: subjective determination is convenient and noninvasive but exists misdiagnosis (the misdiagnosis rate was 27.59% according to the statistic in the study [8]); pathologic confirmation is an accurate golden standard for lung cancer diagnosis, but it is invasive and time-consuming. Therefore, a CT images-based analysis method is highly demanded to be explored to improve the diagnostic accuracy of lung cancer, which can be not only close to the pathologic-level diagnosis but also reduce unnecessary false-positive biopsies or thoracotomy.

**Figure 2:**
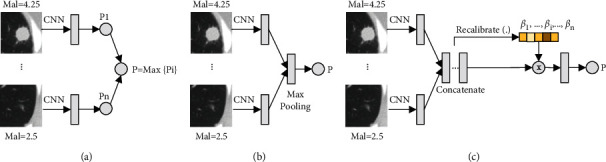
Different types of multi-instance learning (MIL) models. (a) Instance-space MIL. (b) Embedding-space MIL. (c) The proposed recalibrated MIL.

Computer-aided diagnosis methods are exploited to approach invasive pathologic evaluation and improve clinical diagnosis efficiency through automatic analysis of noninvasive CT images [8, 11–13]. In this study, a patient-level lung cancer diagnosis is defined as a multi-instance task, and therefore the prediction of patient-level lung cancer can be formulated as a multi-instance learning (MIL) problem. Multi-instance learning is built on the concept of bag and instances [14]; a patient can be defined as a bag, and the nodules of a patient case can be defined as the instances in the bag. Thus, a multi-instance learning model can be established to predict patient-level lung cancer to be positive or negative, and the bag label probability is fully parameterized by a neural network. Shen et al. [8] proposed a convolutional neural network (CNN)-based instance-space MIL network for the prediction of patient-level lung cancer. As shown in Figure 2(a), the instance-space MIL network first predicts the malignancy probability for each instance (nodule) and then obtains the positive probability of the bag (patient) by calculating the maximum malignancy probability of all instances. This type of instance-space MIL model may introduce additional error to the final bag prediction because its bag label relies on the most positive instance, whereas the probability of malignancy prediction for this most positive instance may be incorrect [15]. Wang et al. [16] revised this type of instance-space MIL to an embedding-space MIL model, which performed a max-pooling operation on the embedding features of all nodules and represented a bag as a single feature embedding (see Figure 2(b)). The embedding-space MIL model determines a joint representation of a bag by aggregating all instance embedding features and then gives a final decision based on the bag representation. This type of embedding-space MIL model can be trained directly with a bag-level classifier instead of the instance-level classifier, avoiding the uncertainty of the malignancy prediction for each nodule compared to the instance-space MIL model. It is suggested that the embedding-space MIL model is more preferable than the instance-space MIL model on MIL benchmarks in previous studies [15, 16]. The superiority of the embedding-space MIL model has also been demonstrated in the patient-level lung cancer prediction in the experiment section of this study.

**Figure 3:**
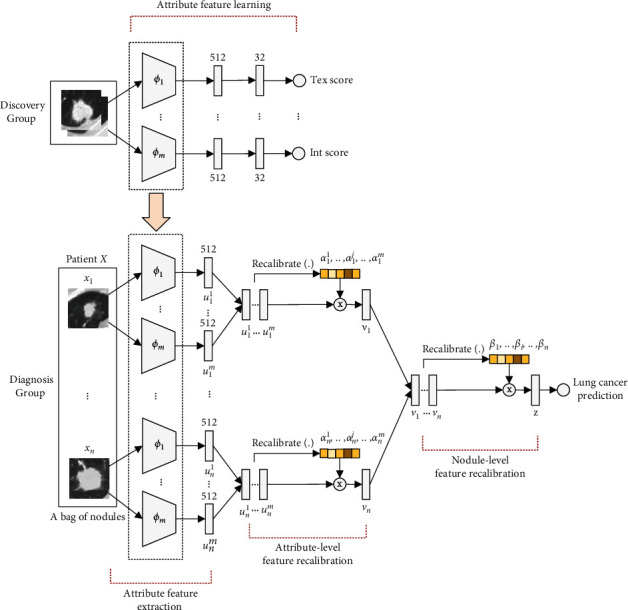
The proposed cascaded-recalibrated multiple instance learning framework based on multiattribute features transfer for pathologic level lung cancer prediction in CT images.

However, the bag labels of the instance-space model and the embedding-space model rely on the most positive individual nodule and the maximum embedding features, respectively. In fact, different nodules of a bag may contribute different information to the prediction of patient-level lung cancer, not just the most positive one or the maximum features. To gain an insight into the contribution of each nodule embedding to the bag target prediction, the weight recalibration for each nodule embedding is involved and an embedding-recalibrated module is introduced to adaptively learn the importance of each nodule in the prediction of patient-level lung cancer, as shown in Figure 2(c). In addition, a nodule also has several attributes, such as texture (tex), lobulation (lob), and sphericity (sph), which are often used to describe various morphological characteristics of nodules. Different attribute features provide different semantic information to nodule feature embeddings, which further enhances the prediction capability of patient-level lung cancer. Likewise, the embedding-recalibrated modules can also be introduced to learn the importance of each attribute for the nodule feature embeddings. Thus, a patient has multiple nodules and each nodule has multiple attributes, which implies a cascaded structure in the patient-level lung cancer prediction, as shown in Figures 1(b) and 1(d). In this study, we proposed a cascaded-recalibrated MIL model by incorporating a cascaded recalibration mechanism at the nodule level and attribute level (see Figure 3)). This cascaded recalibration mechanism enables the MIL model to pay more attention to those important nodules and attributes while suppressing less-useful feature embeddings. The identification of the key nodules and attributes also provides better interpretability for the model decision-making process, which is very important for medical applications in clinic.

The contribution of our study is summarized as threefold. (1) We propose a cascaded-recalibrated MIL model, in which the cascaded recalibration strategy first fuses the informative attribute features into nodule embeddings, and then the key nodule features can be converged into the lung cancer representation to improve the performance of computer-assisted prediction. (2) We also quantitatively analyze the contribution of the nodule and attribute features and reveal the underlying relationship between the confirmed diagnosis and its highly correlated attributes, which demonstrates the robustness and the interpretability of the cascaded-recalibrated MIL model. (3) We validate our proposed MIL model on the public LIDC-IDRI dataset and achieved superior performance on patient-level lung cancer prediction, and this CT image-based computer-aided analysis method has the potential to improve the diagnosis of lung cancer.

## 2. Dataset and Preprocessing

The public Lung Image Database Consortium and Image Database Resource Initiative (LIDC-IDRI) dataset [9] was used in this study to demonstrate the efficacy of the proposed cascaded-recalibrated MIL model for pathologic-level lung cancer prediction in CT images. The LIDC-IDRI dataset contains 1018 cases of lung cancer screening thoracic CT scans with marked-up annotated nodules. Each case includes a series of clinical thoracic DICOM images and an associated XML file that records the results of nodule markings performed by four experienced thoracic radiologists with rigorous reading protocol [9]. The nodules larger than 3 mm in diameter were also scored with 9 types of semantic attributes, i.e., texture, sphericity, malignancy, lobulation, spiculation, margin, calcification, subtlety, and internal structure, which are abbreviated as “tex,” “sph,” “mal,” “lob,” “spi,” “mar,” “cal,” “sub,” and “int,” respectively. Each type of attribute describes different semantic characteristics of the nodules for the diagnostic reference. For example, attribute “tex” indicates whether the nodules appear solid in the CT image, “sph” suggests the roundness of nodule shape, and “mal” stands for the subjective evaluation of malignancy for nodules [17]. All attributes were scored within a range of [1, 5], except for “cal” with a range of [1, 6].

Referring to the nodule size reported in [18, 19], the regions of interest (ROIs) of nodule lesions were cropped into 64 *∗* 64 pixels and transformed into gray-scale images with the HU lung window range of [−1400, 200] as in [13, 20, 21]. In this study, a total of 2632 nodules larger than 3 mm in diameter were extracted and each nodule was rated with 9 types of attribute scores. Each type of attribute score from all radiologists was averaged as the ground-truth scores for training and test when a nodule was rated by more than one radiologist [8, 13, 20–23].

The LIDC-IDRI dataset totally involved 1010 patients, of whom only 117 had pathologically confirmed patient-level lung cancer positive or negative labels. Pathologic confirmation is the golden standard for clinical diagnosis of lung cancer. Based on the presence and absence of the pathologically confirmed diagnosis of lung cancer, we split the 2632 nodules into a diagnosis group and a discovery group at the patient level as in [8, 13]. The diagnosis group includes 117 patients (31 negative and 86 positive) with a total of 349 nodules, and the remaining 2283 nodules are divided into the discovery group. Accordingly, the diagnosis group includes 9 types of attribute scores and one pathologically confirmed lung cancer label, while the discovery group includes 9 types of attribute scores but not the pathologically confirmed lung cancer label.

## 3. Methods

In this section, we introduce our designed cascaded-racalibrated multiple instance deep learning framework for lung cancer prediction by considering the contributions of the attributes and nodules to the final patient-level decision making process. The cascaded-racalibrated MIL model has a CNN architecture that takes a bag (one patient) of input images (nodule lesions), and output one decision: is this patient (bag) lung cancer positive? Since the acquisition of pathologically confirmed lung cancer makers (the target label in MIL model training) often requires invasive operaions such as biopsy or surgery, it is very challenging to obtain sufficient target labels for the training of the cascaded-recalibrated MIL model as well as the typical MIL learning models. In contrast, the attribute scores assessed by radiologists are noninvasive and much more abundant than the pathologic labels. Meanwhile, semantic attributes such as texture, sphericity, and malignancy are highly correlated with the target pathologic labels, and the attribute features can be easily transferred to the target domain. Therefore, we propose a cascaded-recalibrated multiple instance learning framework based on multiattribute features transfer for pathologically level lung cancer prediction in CT images. Specifically, an attribute-specific learning framework is first built to learn attribute features extraction in the discovery group, and then the learned feature extractors are used in the diagnosis group to facilitate attribute-level and nodule-level feature recalibrations in the cascaded-recalibrated multiple instance learning framework, as shown in Figure 3. The proposed learning framework mainly consists of five parts: attribute feature learning, attribute feature extraction, attribute-level feature recalibration, nodule-level feature recalibration, and patient-level lung cancer prediction. It is worth noting that the first part, i.e., attribute feature learning, is trained on the discovery group, and the rest four parts are optimized on the diagnosis group. For simplicity, we denote the discovery group as *D*_*c*_ and the diagnosis group as *D*_*g*_.

### 3.1. Attribute Feature Learning

To learn transferable semantic knowledge from experts, we construct the attribute-specific models to mimic radiologist-like attribute score estimation on nodule level via attribute score regression on the discovery group *D*_*c*_, as shown in the upper part of Figure 3. The attribute-specific model is composed of a feature extration module *ϕ* and a regression module ℛ that simply predicts the scores of nodule attributes. The feature extraction module takes nodule patches as input and learns high-level semantic feature maps. This feature extraction module employs the 18-layer residual network [24] without its final classification layer as the backbone fine-tuned from ImageNet [25]. The regression module is built to provide attribute scores in a range for each nodule based on the high-level deep features from the feature extraction module. This regression module consists of three fully connected layers: 512 neurons as the input layer, 32 neurons as the hidden layer, and 1 neuron as the output, where the first two fully connected layers are applied with ReLU activation, and the output neuron is operated with mean squared error (MSE) loss. For each attribute *s*, *s* ∈ *S*, and *S*={tex,sph,mal,lob,spi,mar,cal,sub,int}, the feature extraction module *ϕ*_*s*_ and the regression module ℛ_*s*_ are jointly trained in an attribute-specific model on *D*_*c*_, and the MSE loss ℒ_reg_ can be minimized in the backward propagation process to predict the attribute scores of nodules as in the following equation:

(1) $$\begin{array}{c} {ℒ}_{\text{reg}}=\underset{{ℛ}_{s},\phi_{s}}{\min}\frac{1}{\left|D_{c}\right|}\sum_{\left(x,y_{s}\right)\in D_{c}}{\left({ℛ}_{s}\left(\phi_{s}\left(x\right)\right)-y_{s}\right)}^{2}, \end{array}$$

where *y*_*s*_ denotes the score of semantic attribute *s* for nodule *x* rated by radiologists. Notablely, each semantic attribute corresponds to a jointly optimized attribute-specific model, and a total of |*S*| feature extraction modules are trained on *D*_*c*_.

### 3.2. Attribute Feature Extraction

The well-trained feature extraction modules *ϕ*={*ϕ*_1_, *ϕ*_2_,…, *ϕ*_*m*_}(*m* ≤ |*S*|) from the discovery group are used to extract attribute features from the diagnosis group. We define one patient (a bag) as *X*, with *n* nodules {*x*_1_, *x*_2_,…, *x*_*n*_} per patient, and each nodule can be described by *m* attributes. For the *j*^*th*^ attribute, the high-level semantic knowledge features *u*_*i*_^*j*^ for the nodule *x*_*i*_ can be extracted by *ϕ*_*j*_ on diagnosis group *D*_*g*_ as in the following equation:

(2) $$\begin{array}{c} u_{i}^{j}=\phi_{j}\left(x_{i}\right),\;i=\left\{1,2,\ldots ,n\right\},j=\left\{1,2,\ldots ,m\right\}. \end{array}$$

### 3.3. Attribute-Level Feature Recalibration

A nodule is commonly described with various attributes, such as texture, calcification, spiculation, and lobulation. These attributes imply that pulmonary nodules have varying degrees of malignancy. Thus, each attribute has a specific contribution to the nodule feature embeddings and further to the decision-making of lung cancer. Here, we propose an attribute-level feature recalibration module according to the importance of each attribute to the representation of nodule features. The mechanism of recalibration for attribute features can elucidate useful attribute features while suppressing nonuseful attribute features by adaptively learning the recalibration coefficients for each attribute of a nodule.

For nodule *x*_*i*_ on the diagnosis group *D*_*g*_, the attribute-level feature recalibration module is proposed to recalibrate the high-level deep features of each attribute. The mechanism of attribute-level feature recalibration aims to explicitly identify the contribution coefficients of the corresponding attributes to the nodule feature embeddings. We calculate the recalibration coefficient *α*_*i*_ for each attribute of nodule *x*_*i*_ inspired by previous work [15, 26]. Suppose that the feature recalibration subnetwork can be represented as Recalibrate(.), the corresponding recalibration coefficient *α*_*i*_^*j*^ for the *j*^th^ attribute of nodule *x*_*i*_ can be formulated as in the following equation:

(3) $$\begin{array}{c} \alpha_{i}^{j}=\frac{\exp\left(\text{Recalibrate}\left(u_{i}^{j}\right)\right)}{\sum_{j=1}^{m}\exp\left(\text{Recalibrate}\left(u_{i}^{j}\right)\right)}, \end{array}$$

where *m* represents the number of attributes involved in the nodules on group *D*_*g*_. The features recalibration subnetwork Recalibrate(.) is constructed with four fully connected layers, and the neurons number of each layer is set as 512, 128, 32, and 1, respectively. The first three fully connected layer is also applied with ReLU activatio, and Recalibrate(.) squeezes the high-level deep features of *m* attributes into one dimension. We compute the nodule embedding *v*_*i*_ as a weighted sum of each attribute embedding *u*_*i*_^*j*^ with its corresponding recalibration coefficient *α*_*i*_^*j*^ as in the following equation:

(4) $$\begin{array}{c} v_{i}=\sum_{j=1}^{m}\alpha_{i}^{j}u_{i}^{j}, \end{array}$$

where the sum of the recalibration coefficients for the attributes of nodule *x*_*i*_ is equal to 1, i.e., ∑_*j*=1_^*m*^*α*_*i*_^*j*^=1.

### 3.4. Nodule-Level Feature Recalibration

A patient can be often found with multiple pulmonary nodules, and each nodule has a specific contribution to lung cancer decision making. To learn the importance of each nodule to the patient-level lung cancer prediction, we also build a nodule-level feature recalibration subnetwork Recalibrate(.) to identify the key nodules of the patient (see the nodule-level feature recalibration part in Figure 3). The nodule-level feature recalibration subnetwork Recalibrate(.) is also constructed with four fully connected layers, and the neurons number of each layer is set as 512, 128, 32, and 1, respectively. The first three fully connected layer is also applied with ReLU activation. The nodule-level Recalibrate(.) squeezes the deep features of *n* nodules into one dimension, i.e., from [*v*_1_,…, *v*_*i*_,…, *v*_*n*_] ∈ *ℜ*^512*∗n*^ to [*β*_1_,…, *β*_*i*_,…, *β*_*n*_] ∈ *ℜ*^*n*^. The corresponding recalibration coefficient *β*_*i*_ for nodule *x*_*i*_ can also be learned by nodule-level Recalibrate(.) as in the following equation:

(5) $$\begin{array}{c} \beta_{i}=\frac{\exp\left(\text{Recalibrate}\left(v_{i}\right)\right)}{\sum_{i=1}^{n}\exp\left(\text{Recalibrate}\left(v_{i}\right)\right)}. \end{array}$$

Similarly, the patient-level lung cancer embedding, denoted as *z*, can be further calculated as a weighted sum of each nodule embedding *v*_*i*_ with its corresponding recalibration coefficients *β*_*i*_ as in the following equation:

(6) $$\begin{array}{c} z=\sum_{i=1}^{n}\beta_{i}v_{i}, \end{array}$$

where the sum of the recalibration coefficients for the nodules of a bag is also equal to 1, i.e., ∑_*i*=1_^*n*^*β*_*i*_ = 1.

### 3.5. Patient-Level Lung Cancer Prediction

We defined the pathologic level lung cancer label (bag label) as *Y*, which can be positive (1) or negative (0). Thus, patient-level lung cancer prediction can be formulated as a binary classification problem. We construct a classification module to fully parameterize the bag prediction probability *P*(*z*). The classification module is composed of three fully connected layers with 512 input neurons, 32 hidden neurons and 1 output neuron, and the first two fully connected layers are activated by ReLU. The training loss for each patient between the bag prediction probability *P*(*z*) and the ground-truth bag label *Y* can be formulated as in the following equation:

(7) $$\begin{array}{c} {ℒ}_{cls}=-\left(1-Y\right)*\;\log\left(1-P\left(z\right)\right)-Y*\;\log\left(P\left(z\right)\right). \end{array}$$

In this way, we gradually construct the cascaded-recalibrated multiple-instance deep learning framework from attribute feature recalibration to nodule-level feature recalibration, and a binary classifier is employed to perform patient-level lung cancer prediction. The proposed cascaded-recalibrated MIL model mirrors the cascaded structure among bag, nodule and attribute, and the two levels of feature recalibrations reflect the importance of attributes and nodules in the bag-level classification, which has the potential to improve the predictive performance of patient-level lung cancer.

### 3.6. Training Scheme

The training scheme is summarized in Algorithm 1. In Step I, a total of 9 attribute-specific models are trained by jointly optimizing the feature extraction module and regression module for each attribute on the discovery group *D*_*c*_. The previous study [8] predicted the patient-level lung cancer based on the only semantic attribute of malignancy. In this study, we seek more important attributes to boost the predictive performance of patient-level lung cancer. Unfortunately, there exists a combination explosion in the selection of 9 attributes to the cascaded-recalibrated MIL model. But fortunately, the associations between each attribute and pathologic level malignancy can be highly different. Therefore, we rank the performance of the 9 semantic attributes in the independent classification of pathologic malignancy with the recalibrated MIL models in step II. The cascaded-recalibrated MIL models are trained in step III by selecting the top-*k* attributes in step II to alleviate the problem of combination explosion. In particular, when only one attribute is involved in the nodule-level recalibration, the recalibration coefficients of this attribute are equal to 1, and thus the attribute embedding is equivalent to the nodule embedding. This is why step II does not include the attribute-level feature recalibration compared to step III.

## 4. Experiments

### 4.1. Experimental Settings

For the robustness of the performance evaluation, the 5-fold cross-validation scheme based on the patient-level data partition was conducted on the training and test for all MIL models, including the instance-space MIL model (see Figure 2(a)), embedding-space MIL model (see Figure 2(b)), recalibrated MIL model (see Figure 2(c)), and cascaded-recalibrated MIL model (see Figure 3). Since all parts of the MIL model are differentiable, we can train these models end-to-end by the stochastic gradient descent (SGD) algorithm. The batch size was set as 1 (=1 bag) [15] and the weight decay was fixed at 1*e* − 4. We set the learning rate as 0.001 and the number of training iterations as 2000.

To tackle the imbalance problem between the positive and negative bags, the positive and the negative bags were randomly selected and alternately used as the input of the MIL models for training. For a fair comparison, all multi-instance models adopted the same network ResNet-18 [24] as the backbone. For the attribute-specific models, the SGD algorithm was also adopted for optimization, with a batch size of 64 and a fixed weight decay of 1*e* − 4. We trained each attribute-specific model for 60 epochs, with a learning rate starting at 0.01 and a factor of 10 reduction per 20 epochs. All experiments were run on a Linux server with 4 NVIDIA Titan X GPUs.

We employ accuracy, AUC, and F1-score for the performance comparison on pathologic level lung cancer prediction in CT images. For the metrics of accuracy and F1-score, if the estimated confidence for any label is less than 0.5, the predicted label is negative. Otherwise, it is positive. In this study, F1 score is evaluated by averaging the negative and positive F1-score.

### 4.2. Performance Evaluation

In this section, we first evaluate whether the radiologists' knowledge of attributes can be properly extracted by the attribute-specific regression models. We trained the models on the discovery group and tested their performance on the diagnosis group.  Table 1 reports the predicted attribute scores by the attribute-specific models and compares them with the corresponding interobserver variation (IB) of radiologists as in [23, 27]. As can be seen in Table 1, for most attributes, the regression performance of our attribute-specific model can be comparable to the assessment of radiologists, which has proved that the attribute-specific regression models based on ResNet-18 [24] were suitable for extracting semantic features of nodules in a limited dataset.

Since most previous studies predict the pathologic level of lung cancer in CT images by transferring from the attribute malignancy (mal), we first evaluate different types of models on the attribute malignancy.  Table 2 reports the predictive performance of the higher-order transfer [13], instance-space MIL, embedding-space MIL, and our recalibrated MIL model on patient-level lung cancer based on the attribute malignancy (mal) transfer. As the lung cancer was confirmed diagnosed at the pathologic level in this study, we simply defined the patient-level lung cancer prediction as pathologic malignancy (pmal) prediction. Since the four models are evaluated with the 5-fold cross-validation scheme, the mean ± standard deviation statistics of accuracy, AUC and f1-score are reported in Table 2. The models of instance-space MIL, embedding-space MIL, and recalibrated MIL are built on the concept of patient-level MIL, whereas the higher-order transfer model belongs to a nodule-level non-MIL method. As can be seen in Table 2, the instance-space MIL, embedding-space MIL, and recalibrated MIL models perform much better than the higher-order transfer model on the metrics of accuracy, AUC, and f1-score, which suggests that the patient-level MIL methods are superior to the nodule-level non-MIL method. This is because the higher-order transfer method assumes that the label of each nodule in the bag is the same as the bag label. In fact, the labels of nodules may be inconsistent with the label of the bag, and even the labels of some nodules are uncertain, which is introduced additional errors into the pmal prediction by the nodule-level non-MIL method. By contrast, the models of instance-space MIL, embedding-space MIL, and recalibrated MIL, aimed at the patient-level pmal prediction, are demonstrated to be more reliable. It also can be found in Table 2 that the embedding-space MIL and the recalibrated MIL methods perform better than the instance-space MIL. This suggests that the weak-supervised MIL has better performance when converted to the bag-level supervised MIL. The recalibrated MIL model outperforms the embedding-space MIL model, which demonstrates that the patient-level fully-supervised MIL model introduced within recalibration mechanism can further improve the predictive performance on the target pmal. This suggests that the recalibration-based MIL is more adaptive in feature learning than the embedding-space MIL.

In view of the superiority of recalibrated MIL model on lung cancer prediction based on the attribute malignancy transfer, we explore more attributes transfer for improving the predictive performance on the target pmal, as in Table 3. To illustrate the associations between pmal and various attributes, we also rank the 9 attributes by the f1-score. For example, attribute “tex” gets the first place, which achieves the best performance in terms of f1-score with 0.813 ± 0.037. The rank order of attributes shown in Table 3 was basically consistent with the previous clinical study [28]. The top-one attribute texture (tex) indicates whether the nodule appears nonsolid, part-solid, and solid in the CT image, where the subsolid nodules that may have purely ground-glass attenuation, partly solid, or mixed solid and ground-glass attenuation are highly associated to the subtypes of lung adenocarcinomas. The likelihood of malignancy (mal) is an important clinical consideration because the malignancy of nodule is highly associated with the risk of developing lung cancer. Morphologic characteristics such as sphericity (sph), lobulation (lob), spiculation (spi), and margin (mar) contour are also useful in the evaluation of nodule malignancy potential. The attribute calcification (cal) represents the calcification pattern of the nodule, and if the nodule is diffusely calcified, it has a high likelihood of being benign. The subtlety (sub) indicates whether the nodule is easy to identify, which may be largely affected by the experience and subjectivity of radiologists. The term “internal structure (int)” specifies that the internal nodule can be soft tissue, fluid, fat, or air, while the LIDC-IDRI dataset consists almost exclusively of soft tissue patterns, resulting in the worst performance on “Int ⟶ Pmal”. The ranking of the attributes implies the different importance of each attribute in providing semantic information for the pmal prediction and also alleviates the problem of combination explosion when selecting the attributes for the cascaded-recalibrated MIL model.

Table 4 reports the predictive performance of our cascaded-recalibrated MIL model on target pmal with the top-*k* attribute sources transfer, where *k*={1,2,…, 9}. The top-*k* sources selected from the ranked attributes in Table 3 are considered as the most efficient combination among the *k* attributes. As can be observed in Table 4, the performance of the cascaded-recalibrated MIL model improves with the increase of the attribute dimension at the beginning but decreases after reaching the performance peak at the combination of “tex,” “sph,” and “mal”. This may be because the increasing source attributes may contain less complementary information for solving the target task. The attribute source dimension is increased without increasing the nodule instances in the feature vector, and the dimensionality of the feature space becomes sparser and sparser which forces the cascaded-recalibrated MIL model to be overfitted by loosing generalizing capability. We also list the runtime of the proposed cascaded-recalibrated MIL model during training and test phases in Table 4. The training time shows the time cost of totally 5-fold training with respect to different number of sources. The test time indicates the average predicted time for each patient case. As can be observed in the last column in Table 4, the average predicted time per case is almost in milliseconds during the test phase, and therefore it is possible to run the proposed MIL model in real-time applications.

We also compare our cascaded-recalibrated MIL model with the previous studies [8, 11–13], as well as the radiologists' ratings. As can be seen in Table 5, the results of the higher-order transfer model show that multiattribute sources combination (tex + diam + lob) could improve the predictive performance of pathologic malignancy, which can be comparable to the radiologists. The proposed cascaded-recalibrated MIL model in this study based on a single attribute source like “mal,” “sph,” or “tex” outperforms the previous studies, demonstrating the important role of nodule-level recalibration in the patient-level lung cancer prediction. Likewise, for our cascaded-recalibrated MIL model, a combination of multiple attribute sources, such as “tex + sph” and “tex + sph + mal,” performs much better than the single attribute source, which suggests that attribute-level and nodule-level cascaded recalibration could significantly boost the predictive performance of pathologic malignancy by using CT images.

**Figure 4:**
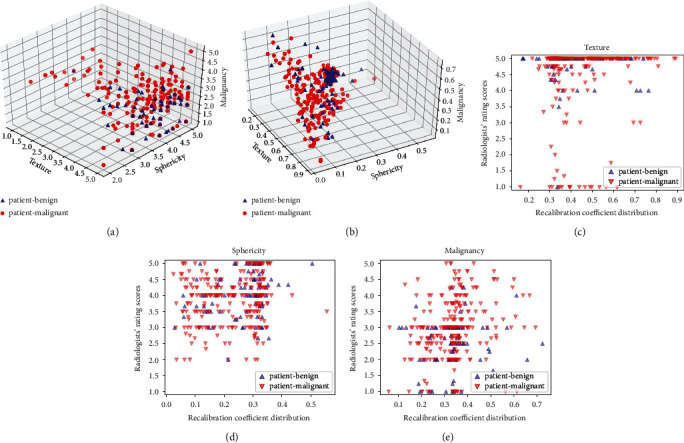
The 3D distribution for the radiologists' rating scores (a) and attribute-level recalibration coefficients (b), respectively, as well as the 2D correlation distribution between the recalibration coefficients and the rating scores for the attributes tex (c), sph (d), and mal (e), respectively.

**Figure 5:**
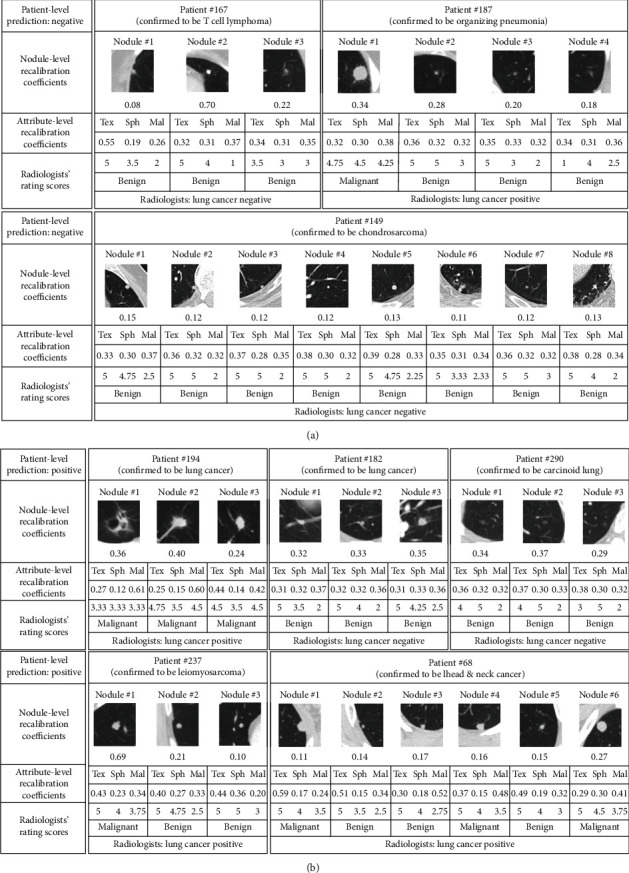
The patient-level decision-making for lung cancer negative (a) and positive (b) examples with the combination of attribute “tex,” “sph,” and “mal” by our cascaded-recalibrated MIL model as well as radiologists. (a) Patient-level decision-making for lung cancer negative examples. (b) Patient-level decision making for lung cancer positive examples.

For the best-performing case (Tex + Sph + Mal ⟶ Pmal) from the cascaded-recalibrated MIL model, we visualize the 3D distribution of the radiologists' rating scores and recalibration coefficients (see Figures 4(a) and 4(b)), respectively, and plot the 2D projection distribution for the attributes tex, sph, and mal referring to the recalibration coefficients and the rating scores (see Figures 4(c)–4(e)). As can be observed from Figures 4(a) and 4(b), radiologists' rating scores and the recalibration coefficients are widely distributed in patient-level negative and positive cases. This suggests that our model can capture diverse semantic dependencies and adaptively assign semantic-dependent weights to the nodules to better identify the negative or the positive for patient-level lung cancer. To intuitively show the associations between the rating scores and the recalibration coefficients, we also plot the 2D projection distribution of the recalibration coeffecients with the radiologists' rating scores for the attributes tex, sph and mal, respectively, as shown in Figures 4(c)–4(e). We also show the patient-level decision-making process in Figure 5 for several lung cancer negative and positive examples with the combination of attribute “tex,” “sph,” and “mal” by the cascaded-recalibrated MIL model as well as the radiologists.

Figure 5(a) shows three negative cases, i.e., patient #167, #187, and #149, all of which are confirmed to be lung cancer negative. Radiologists diagnosed that all nodules in patients #167 and #149 were relatively benign (Mal ≤ 3), which was consistent with the pathologic confirmation and our cascaded-recalibrated MIL model. For patient #167, nodule #2 was rated at a low-level malignancy (Mal = 1) by radiologists, and our model also indicated that nodule #2 had a large nodule-level recalibration coefficients (0.70) with the biggest contribution to the final prediction. In contrast, the coefficients of the nodules in patient #149 had a more uniform distribution, ranging from 0.11 to 0.15, which suggested that there was no significant difference in the contribution of each nodule to the decision-making of the model. Our model predicted patient #187 to be negative and assigned a relatively large nodule-level recalibration coefficient to nodule #1, whereas the radiologists had rated a high level of malignancy for nodule #1 (Mal = 4.25), and this patient was considered to be positive by radiologists. In fact, patient #187 turned out to be organizing pneumonia and confirmed to be lung cancer negative by surgical resection.

Figure 5(b) reports five positive cases of patient-level lung cancer, where patients #194, #182, and #290 were confirmed as the primary lung cancer, and patients #237 and #68 turned out to be metastatic lung cancer. For patient #194, the subjective judgment of radiologists on each nodule was relatively malignant, which was consistent with the biopsy examination. Meanwhile, our model also accurately predicted patient #194 as lung cancer positive with relatively high attribute-level recalibration coefficients on malignancy for each nodule (0.61, 0.60, 0.42). Both patients #182 and #290 were assessed to be relatively benign (Mal ≤ 2.5) by radiologists. In particular, there are very subtle nodules in patient #290, which appear to be very small spots and must be carefully observed in the center of the image patches. Our model was still able to accurately predict that patients #182 and #290 were lung cancer positive, with a relatively uniform distribution of recalibration coefficients on the two levels of nodules and attributes. Nodule #1 in patient #237 has a popcorn-like appearance and was distinctly malignant, to which our model adaptively assigned a larger coefficient (0.69). For the patient #68, the coefficients difference on the first five nodules, i.e., from nodule #1 to #5, was not significant (ranging from 0.11 to 0.17), while the recalibration coefficients on nodule # 6 were slightly larger (0.27), indicating a higher degree of malignancy on nodule #6 than the remaining five nodules. In summary, this study's results demonstrated that the proposed cascaded-recalibrated MIL model not only could predict lung cancer at the patient-level more accurately but also successfully detect the key nodules and attributes that were more interpretable for the model decision.

## 5. Conclusion

In this study, we proposed a cascaded-recalibrated multiple instance learning framework based on multiattribute features transfer for improving lung cancer prediction in CT images. The cascaded-recalibrated MIL model was progressively constructed from the multiattribute embedding recalibration to the nodule embedding and then from the multinodule embedding recalibration to the patient-level lung cancer embeddings. This two-level cascaded recalibration could quantitatively reflect the importance of the nodules and the attributes in the patient-level lung cancer prediction. Since there were too many combinations of the 9 semantic attributes, it was a huge project to run all combinations to verify the effectiveness of the proposed method. In order to solve the problem of combination explosion, we ranked the performance of these 9 attributes in the independent classification of pathologic malignancy and took some of the best performers as the input of the proposed MIL model. This resulted in not all combinations being taken as inputs. Nevertheless, the experiment demonstrated that the proposed MIL model based on the current attribute combinations performed significantly better than the previous studies, as well as the radiologists. This indicated that the current combinations of the attributes could verify the effectiveness of the proposed MIL model to some extent. Additionally, we also showed the attribute-level and nodule-level recalibration coefficients during the patient-level decision-making process, and the detected key attributes and nodules were more interpretable for the model decision.

## Figures and Tables

**Algorithm 1:**
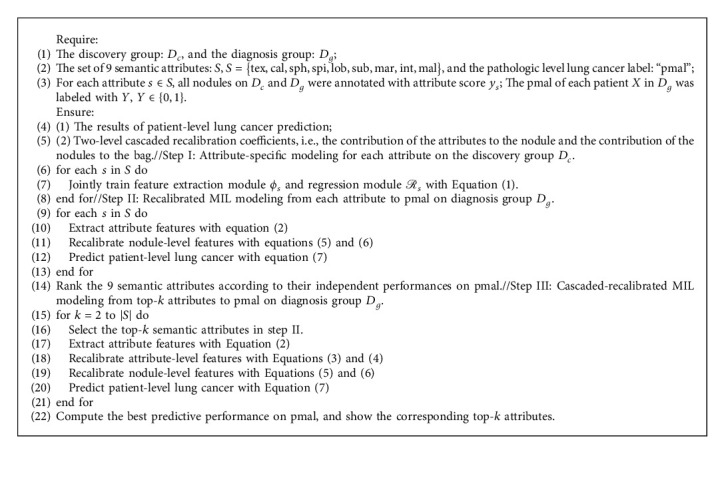
Cascaded-recalibrated multiple instance learning based on multiattribute features transfer for pathologic level lung cancer prediction in CT images.

**Table 1:** Predictive performance of our attribute-specific models and the radiologists' on nine semantic attributes, evaluated by mean absolute error (MAE). The abbreviation “IB,” which indicates the interobserver variation, is calculated based on all possible pairs of scores given by radiologists as in [23, 27].

MAE	Sub	Int	Cal	Sph	Mar	Lob	Spi	Tex	Mal
IB	0.70	**0.02**	**0.20**	0.88	0.67	0.83	0.67	**0.22**	0.90
Attribute-specific model	**0.65**	0.09	0.24	**0.51**	**0.54**	**0.51**	**0.42**	0.41	**0.44**

The bold values mean the superiority to others.

**Table 2:** Performance comparison of lung cancer prediction (pmal) by transferring from attribute malignancy in terms of the models of higher-order transfer learning, instance-space MIL, embedding-space MIL, and the proposed recalibrated MIL.

Mal ⟶ Pmal	Accuracy	AUC	F1-score
Higher-order transfer [13]	0.702 ± 0.050	0.669 ± 0.045	0.644 ± 0.035
Instance-space MIL	0.820 ± 0.044	0.793 ± 0.059	0.780 ± 0.054
Embedding-space MIL	0.820 ± 0.034	0.800 ± 0.043	0.783 ± 0.031
Recalibrated MIL (ours)	**0.837** ± **0.035**	**0.830** ± **0.021**	**0.800** ± **0.037**

The bold values mean the superiority to others.

**Table 3:** Performance of lung cancer prediction (pmal) for the proposed recalibrated MIL model by transferring from each attribute.

Recalibrated MIL	Accuracy	AUC	F1-score	Ranking
Tex ⟶ Pmal	0.846 ± 0.037	**0.849** ± **0.027**	**0.813** ± **0.037**	1
Sph ⟶ Pmal	**0.848** ± **0.059**	0.831 ± 0.080	0.803 ± 0.073	2
Mal ⟶ Pmal	0.837 ± 0.035	0.830 ± 0.021	0.800 ± 0.037	3
Lob ⟶ Pmal	0.822 ± 0.065	0.818 ± 0.050	0.797 ± 0.058	4
Spi ⟶ Pmal	0.838 ± 0.061	0.811 ± 0.056	0.796 ± 0.075	5
Mar ⟶ Pmal	0.813 ± 0.061	0.819 ± 0.038	0.789 ± 0.056	6
Cal ⟶ Pmal	0.803 ± 0.044	0.796 ± 0.032	0.768 ± 0.038	7
Sub ⟶ Pmal	0.802 ± 0.048	0.775 ± 0.086	0.767 ± 0.057	8
Int ⟶ Pmal	0.794 ± 0.076	0.704 ± 0.079	0.743 ± 0.064	9

The bold values mean the superiority to others.

**Table 4:** Performance of cascaded-recalibrated MIL model based on the top-*k* attribute sources transfer.

Top-*k*	Sources ⟶ Target(cascaded-recalibrated MIL)	Accuracy	AUC	F1-score	Runtime	
					Training	Test (s)
Top-1	Tex ⟶ Pmal	0.846 ± 0.037	0.849 ± 0.027	0.813 ± 0.037	6 min 31 s	0.0035
Top-2	Tex + Sph ⟶ Pmal	0.863 ± 0.051	0.867 ± 0.070	0.834 ± 0.062	9 min 4 s	0.0046
Top-3	Tex + Sph + Mal ⟶ Pmal	**0.880** ± **0.032**	**0.877** ± **0.036**	**0.849** ± **0.037**	12 min 0 s	0.0066
Top-4	Tex + Sph + Mal + Lob ⟶ Pmal	0.847 ± 0.056	0.842 ± 0.053	0.817 ± 0.066	15 min 17 s	0.0083
Top-5	Tex + Sph + Mal + Lob + Spi ⟶ Pmal	0.846 ± 0.020	0.82 ± 0.0460	0.800 ± 0.031	18 min 27 s	0.0098
Top-6	Tex + Sph + Mal + Lob + Spi + Mar ⟶ Pmal	0.837 ± 0.065	0.833 ± 0.065	0.808 ± 0.071	21 min 42 s	0.0103
Top-7	Tex + Sph + Mal + Lob + Spi + Mar + Cal ⟶ Pmal	0.829 ± 0.028	0.821 ± 0.056	0.791 ± 0.036	24 min 30 s	0.0133
Top-8	Tex + Sph + Mal + Lob + Spi + Mar + Cal + Sub ⟶ Pmal	0.828 ± 0.041	0.821 ± 0.043	0.798 ± 0.047	28 min 16 s	0.0146
Top-9	Tex + Sph + Mal + Lob + Spi + Mar + Cal + Sub + Int ⟶ Pmal	0.803 ± 0.020	0.816 ± 0.013	0.770 ± 0.028	31 min 27 s	0.0161

The bold values mean the superiority to others.

**Table 5:** Performance comparison of lung cancer prediction (pmal) in terms of accuracy and AUC.

Method	Accuracy	AUC
Radiologists' ratings (Mal ⟶ Pmal)	0.7106	0.7621
DARS [11]	0.7501	—
DRS [12]	0.7752	—
CNN nodule(Mal ⟶ Pmal) [8]	0.6538	0.63
CNN-MIL (Mal ⟶ Pmal) [8]	0.7069 ± 0.02	0.66 ± 0.03
Higher-order transfer (Mal ⟶ Pmal) [13]	0.7019 ± 0.05	0.6688 ± 0.05
Higher-order transfer(Tex ⟶ Pmal) [13]	0.7677 ± 0.07	0.7293 ± 0.07
Higher-order transfer(Tex + Diam + Lob ⟶ Pmal) [13]	0.8194 ± 0.02	0.7533 ± 0.05
Recalibrated MIL(Mal ⟶ Pmal) (ours)	**0.837** ± **0.035**	**0.830** ± **0.021**
Recalibrated MIL(Sph ⟶ Pmal) (ours)	**0.848** ± **0.059**	**0.831** ± **0.080**
Recalibrated MIL(Tex ⟶ Pmal) (ours)	**0.846** ± **0.037**	**0.849** ± **0.027**
Cascaded-recalibrated MIL(Tex + Sph ⟶ Pmal) (ours)	**0.863** ± **0.051**	**0.867** ± **0.070**
Cascaded-recalibrated MIL(Tex + Sph + Mal ⟶ Pmal) (ours)	**0.880** ± **0.032**	**0.877** ± **0.036**

The bold values mean the superiority to others.

## Data Availability

The dataset LIDC-IDRI used in this study is a public dataset and can be accessed through this link: https://wiki.cancerimagingarchive.net/display/Public/LIDC-IDRI.
